# Supplementary material for: The genome sequence of a sawfly Macrophya annulata (Geoffroy, 1785)
Source: Wellcome Open Res. 2024 Jul 18;9:388. [Version 1] doi: 10.12688/wellcomeopenres.22612.1 (PMC11406137; doi:10.12688/wellcomeopenres.22612.1)
Supplement: Peer review history

# Reviewer report: Reviewer response for version 1

**DOI:** 10.21956/wellcomeopenres.24911.r96562

**Published:** 2024-09-16

**Authors:** Arka Mukherjee

**Affiliations:** 1 Zoological Survey of India, Kolkata, West Bengal, India

This paper describes the complete genome sequence of an effective spider-hunting wasp *Macrophya annulata *(Geoffroy, 1785). The distribution of the species is Palearctic region. The article purely focused on the complete genome sequences of the sawfly. All the assembly and annotation of the genome are executed properly. In the background section, there is a minor mistake present - “This complete **gene** sequence will help our understanding of the phylogeny of this group.” Here the ‘gene’ should be replaced by **genome**. As the author mentioned in the manuscript, two haplotypes are present, and if both are deposited, then phasing is not required. This complete genome enriches the global biodiversity genomics and also helps to study  the evolution of sawflies.

Are sufficient details of methods and materials provided to allow replication by others?

Yes

Is the rationale for creating the dataset(s) clearly described?

Yes

Are the datasets clearly presented in a useable and accessible format?

Yes

Are the protocols appropriate and is the work technically sound?

Yes

Reviewer Expertise:

Taxonomy, DNA barcoding, Mitochondrial genome, Phylogeny, Diptera

I confirm that I have read this submission and believe that I have an appropriate level of expertise to confirm that it is of an acceptable scientific standard.

# Reviewer report: Reviewer response for version 1

**DOI:** 10.21956/wellcomeopenres.24911.r95320

**Published:** 2024-08-29

**Authors:** Marko Prous

**Affiliations:** 1 University of Tartu, Vanemuise, Tartu, Estonia

The paper reports high quality chromosome level genome of a sawfly *Macrophya annulata*. The structure of the paper and methods are standard in this journal.

 There seem to be some errors:

 1) In the Background section you write "This complete gene sequence will help our understanding of the phylogeny of this group."

 I guess you mean "genome sequence" instead of "gene"? Please correct.

 2) You stated that the sex of the sequenced specimen is a male. As I did not find a second deposited haplotype the sentence "While not fully phased, the assembly deposited is of one haplotype. Contigs corresponding to the second haplotype have also been deposited." should be corrected.

 Check this and if necessary correct that there is only one haplotype because the sample is haploid. No phasing needed. Seems you have have copy-pasted the text from previous publications.

Are sufficient details of methods and materials provided to allow replication by others?

Yes

Is the rationale for creating the dataset(s) clearly described?

Yes

Are the datasets clearly presented in a useable and accessible format?

Yes

Are the protocols appropriate and is the work technically sound?

Yes

Reviewer Expertise:

Sawfly taxonomy, phylogenetics, and genomics.

I confirm that I have read this submission and believe that I have an appropriate level of expertise to confirm that it is of an acceptable scientific standard.
